# Filicide in Austria and Finland - A register-based study on all filicide cases in Austria and Finland 1995-2005

**DOI:** 10.1186/1471-244X-9-74

**Published:** 2009-11-21

**Authors:** Hanna Putkonen, Sabine Amon, Maria P Almiron, Jenny Yourstone Cederwall, Markku Eronen, Claudia Klier, Ellen Kjelsberg, Ghitta Weizmann-Henelius

**Affiliations:** 1 Vanha Vaasa hospital, PO Box 13, 65381 Vaasa, Finland; 2 Medical University of Vienna, Department of Child and Adolescent Psychiatry, Währinger Gürtel 18-20, A-1090 Vienna, Austria; 3 University of Vienna, Faculty of psychology, Liebiggasse 5, A-1010 Wien, Austria; 4 BFPO 5536, Foreign and Commonwealth Office, West End Road, Ruislip HA4 6EP, UK; 5 Centre for Violence Prevention, Karolinska Institute, Box 23000, 104 35 Stockholm, Sweden; 6 Oslo University Hospital, Ullevaal Department of Psychiatry, Gaustad Building No 7, N-0403 Oslo, Norway

## Abstract

**Background:**

Filicide is the tragic crime of murdering one's own child. Previous research has found that the offending parents are commonly depressed and that suicide is often associated as an actual act or an intention. Yet, filicide is an underreported crime and previous studies have been strained with methodological problems. No comprehensive international studies on filicide have been presented in the literature until now.

**Methods:**

This was a descriptive, comprehensive, register-based study of all filicides in Austria and Finland during 1995-2005. Filicide-suicide cases were also included.

**Results:**

Most of the perpetrators were the biological mothers; in Austria 72%, in Finland 52%. Suicide followed filicide either as an attempt or a fulfilled act in 32% and 54% of the cases in Austria and Finland, respectively. Psychotic mood disorders were diagnosed for 10% of the living perpetrators in Austria, and 12% in Finland. Non-psychotic depression was diagnosed in 9% of surviving perpetrators in Austria, 35% in Finland.

**Conclusion:**

The data from the two countries demonstrated that filicide is such a multifaceted and rare phenomenon that national data from individual countries seldom offer sufficient scope for its thorough study. Further analyses are needed to produce a complete picture of filicide.

## Background

The estimated global rate of child homicide is 1.92 for girls and 2.93 for boys in the age group 0-17 years per 100,000 inhabitants [1]. However, infant homicide figures are not specified in these statistics and child homicide rates are usually considered underestimates [2]. Filicide is the deliberate act of a parent killing her/his own child. It is frequently subcategorized as infanticide when the child is younger than one year and neonaticide when the child has been born not more than 24 hours earlier. Filicide is clearly an exceptional form of homicide. It has been noted that rates of infanticide correspond to suicide rates rather than to murder rates [3]. Undeniably, both attempted and fulfilled suicide often follow filicide [4,5]. Furthermore, there are contrasting findings as to whether mothers or fathers have a higher propensity to commit filicide [6-9]. Previous studies have demonstrated an association between filicide and parental psychiatric illness, specifically major depression with psychotic features [4,5,10]. On the other hand, psychopathy does not seem to be associated with filicide [11]. In the present century, several reviews on filicide have been published [4,5,12-14]. Yet, a dearth of knowledge still exists. Filicide being a comparatively rare event, the data of previous studies have commonly been limited, examined only as subgroups of filicide, or burdened with other methodological problems. Quite often such studies have suffered from non-uniform definitions as well as non-transparent or non-comprehensive material leading to non-generalisable findings. To our knowledge, no previous comprehensive international studies on the subject of filicide exist.

In order to tackle the methodological problems and limited statistical significance of earlier filicide research, a larger cohort was needed. Therefore, Austria and Finland coordinated their efforts to explore the phenomenon with an international study. The filicide-suicide and neonaticide categories were included to gain maximum comprehensiveness. The key objectives of the collaboration are to recover in-depth information on the actual prevalence of filicide, psychiatric morbidity of the perpetrators, gender-related issues, and to identify putative new subgroups of filicide using a large sample. Most importantly, this should lead to a better insight into potential preventive actions. In this first article we aimed to illustrate our data collection procedure, highlight some national features related to filicide and explain basic results on psychiatric and crime scene variables. A primary interest was to investigate what kind of differences arose between the two countries in order to gain some clarification as to whether filicide is a phenomenon that can be studied with international material or should remain area/culture specific. A minor aim was to examine if non-biological and biological parents can be studied in joined analyses.

## Methods

The material of the present study was register-based, comprehensive, and nationwide in Austria and Finland covering all filicides between 1995 and 2005, inclusive. Both countries have a tradition of reliable registers and they have been successfully used for study purposes in the past [15-19]. Registration coverage for births and deaths is over 90% in both countries [20]. In Finland and Austria, almost no victims of homicide remain unknown by the police [21] [unpublished data created by Statistics Austria]. Hence, the rate of hidden criminality for homicide is low in both countries. During 1995-2005, the homicide clearance rate was high in both countries (Austria 90%, Finland 92%) [22,23]. Furthermore, an appreciative percentage of homicide offenders undergo a forensic psychiatric examination: in Finland 85% [24], in Austria 60-90% [Schanda, H. personal communication 2008]. In Austria, there is no central agency to gather data on examinations, hence the range of estimate. In each case the individual court assesses the utility of a forensic examination and court order. Any matters indicating possible mental health matters (psychiatric history, exceptional crime scene circumstances or victim groups etc.) will lead to an examination. Some general information on each country associated with filicide is reported in Table 1.

**Table 1:** Core Health Indicators from WHO sources for Austria and Finland and the USA

**Indicator**^a^	Austria	Finland	USA
Population in millions, 2006	8.3	5.3	302.8
Population median age (years), 2006	40	41	36
Total fertility rate, women	1.4	1.7	2.0
Gross national income per capita international $	28350	23920	35190
Population in urban areas (%)	66	61	79
General government expenditure on health as percentage of total government expenditure	14.7	10.2	19.5
Per capita recorded alcohol consumption (liters of pure alcohol) among adults, 2003	11.08	9.31	8.61
Suicide mortality per 100,000 population, women	10.4	10.9	4.0
Suicide mortality per 100,000 population, Men	29.8	34.6	17.1
Homicide mortality per 100,000 population, < 1 year -- girls	10.5	0	7.4
Homicide mortality per 100,000 population, < 1 year -- boys	2.5	0	9.8
Homicide mortality per 100,000 population, 1--4 years -- girls	0.6	0.9	2.1
Homicide mortality per 100,000 population, 1--4 years -- boys	1.1	0.8	2.5

^a ^WHO Core Health Indicators. http://www.who.int/whosis/en/ and WHO Mortality database http://www.who.int/healthinfo/morttables/en/index.html both accessed November 12, 2008. All statistics are for 2000 unless otherwise marked. The year 2000 was chosen because our data covers 1995--2005. Statistics for the USA are given to enhance comparison.

In both Austria and Finland legislation is based on written law and statutes, not on common law. In both countries filicide is commonly tried as murder, manslaughter or involuntary manslaughter, depending on intentionality, but in Finland, also on brutality of the crime. However, neonaticide has a separate paragraph in both countries and the legislation states the following: The crime committed by a woman, who in a postpartum state of exhaustion or anxiety kills her child, will be ruled neonaticide, and she will be subject to not fewer than four months imprisonment in Finland, one year in Austria, and not more than four years in Finland, five years in Austria. Attempted neonaticide is criminalised as well, but paternal neonaticide is not covered in either of the germane laws. In some countries (e.g. Canada), the Criminal Code provides for a defence of "Infanticide", referring to similar concepts.

In both Austria and Finland, perpetrators will be tried with full criminal responsibility unless proven otherwise. The courts decide if a forensic psychiatric examination is needed to assess criminal responsibility. In Finland, before such a decision, the perpetrator must first be ruled culpable. The processes of the forensic psychiatric examinations share several similarities in both countries. In Finland, a national agency operating under a ministry (The Ministry of Social Affairs and Health) controls the quality of examinations and hands down its own opinion on the conclusions. Moreover, forensic psychiatric examinations are inpatient assessments lasting six weeks on average in Finland, and two months in Austria. They comprise data gathered from various sources (medical, educational, and social services records as well as family and other informants), psychiatric assessment, standardised psychological tests, and continuous observation by hospital staff. In Finland they also include interviews by a multiprofessional team and a physical evaluation. Yet, the Austrian forensic assessments are not as systematic as in Finland. The examinee is met by a forensic psychiatrist and sometimes by a clinical psychologist, who conduct one or several interviews. The final forensic psychiatric report includes an assessment of the level of criminal responsibility and possibly a psychiatric diagnosis. Diagnoses made during the examinations were based on DSM-III-R criteria in Finland and ICD-9 in Austria until 1996, when ICD-10 became the official classification. In addition, DSM-IV has been commonly used. The general quality and reliability of the forensic psychiatric examinations are considered high by both courts and scientists [15,25].

### Procedure

The current paper is based on total population material from both countries. It was part of a larger study project on filicide, the European Collaboration for the Understanding of Filicide. We gathered data on all filicides occurring between 1995 and 2005, inclusive.

The working definition for filicide was: a parent's killing of her/his child. Parent was defined as biological, step or foster parent. A parental relationship or longstanding live-in relationship had to exist in order to be considered as a stepparent. We gathered coroner reports and death certificates from Coroner Institutions of Austria and from Statistics Finland for information on children who had died under the age of 18. There were 150 child homicide victims in Austria and 88 in Finland. Of these, filicide victims numbered 86 and 66, respectively. Victims died in 74 events in Austria and in 50 in Finland.

Based on the information of Austrian coroner reports, requests were sent for data from the Department of Justice for all court files, which included all information relevant for the present study. Similarly, using Finnish death certificates, we requested police files containing the criminal reports of the relevant cases in order to assemble information on the perpetrators. We then searched the following sources: the National Finnish Hospital Discharge register held by The National Research and Development Centre for Welfare and Health (STAKES) for the perpetrators' treatment history, the registers of The National Authority for Medicolegal Affairs (NAMA) for their forensic psychiatric examination reports, and The Legal Register Centre for their criminal records, i.e., the rulings in the cases. In short, we gathered all available register based data associated with the juridical and health related processes on all filicide cases during the study period.

The study included 519 variables, most of them dichotomic at nominal level. Study variables were chosen based on previous literature and covered demographic information, situational factors, possible motives, social, occupational, criminal, psychiatric, and developmental history of the perpetrator as well as that of the victim(s), past and present family matters, possible pregnancy concerns, and legal issues (criminal responsibility, juridical outcome). Continuing discussion was the rule throughout the data collection period to ensure mutual consistency of the coding of each variable. Inter-rater reliability was calculated using two separate cases sent from a UK collaboration partner. There were three raters involved, one in Austria and two in Finland. We selected the following variables for the calculation of interrater agreement: motive and method of offence, relationship between perpetrator and victim, mental health treatment of perpetrator since adulthood and at the time of the offence, perpetrator's intoxication at the time of the offence, possible victimisation of the child by the perpetrator, possible loss of custody, and the juridical decision. The interrater agreement was assessed by computing the Kappa [26,27], which registered maximum agreement (1.0) on 28 of the 58 variables, but the agreement was lower (.33) on the other variables due to lack of information on the cases. In Finland, the agreement between the two raters was κ = 0.82. All coefficients were sound according to the guidelines provided by Cicchetti [28]. In the present study we reported the basic variables related to the victim, offender and the crime scene, including demographic and psychiatric variables.

Chi-square analysis and Fisher's Exact Test were used to compare differences in frequencies between the countries. The Independent Samples t-test was used to compare differences in mean of perpetrators' ages and Mann-Whitney U-test to compare victims' ages. Findings were considered significant if *p *< 0.05.

Ethical approval was granted by the following agencies: Austria: Austrian Ethics Commission, The Department of Justice, Medical University of Vienna; Finland: Ministry of Social Affairs and Health, Ministry of the Interior, The Office of the Data Protection Ombudsman, Ethics Committee for Paediatrics, Adolescent Medicine and Psychiatry of Helsinki University Central Hospital.

## Results

**Figure 1:**
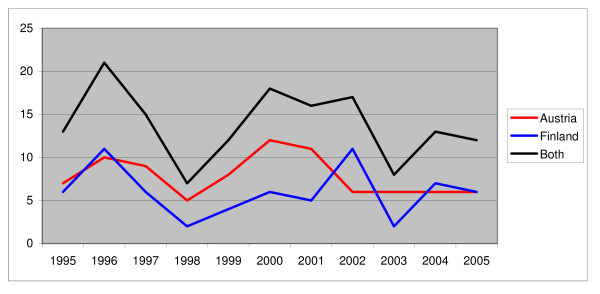
**Temporal distribution of cases**.

**Figure 2:**
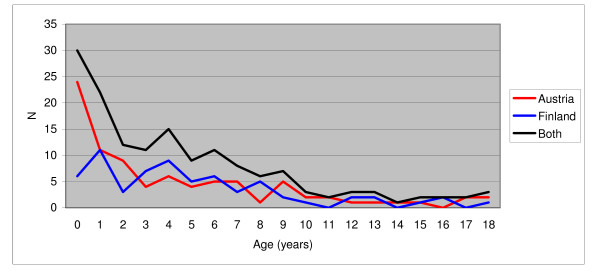
**Age distribution of the victims**.

Austria had 86 filicide victims and Finland had 66, which equal 5.2 per 100,000 inhabitants and 5.9 per 100,000 inhabitants, respectively (Table 2). The temporal distribution of cases is shown in Figure 1. In Austria there were 74 perpetrators, in Finland 50. There were less male perpetrators in Austria than in Finland (27% vs. 48%, p < 0.05, Fisher's exact test). The mean age of the perpetrators in the two countries did not differ significantly (t = -1.494, p = 0.138) nor did the mean age of the victims (z = -1.745, p = 0.81). However, there were significantly more neonaticides among the filicide cases in Austria than in Finland (27% vs. 8%, p < 0.01, Fisher's exact test). The age distribution of the victims is illustrated in Figure 2. Further information on victims and perpetrators is presented in Table 2.

**Table 2:** Filicide in Austria and Finland 1995--2005, victim and perpetrator information

	Austria	Finland	p
Children (<18) killed by homicide	<150 ^a^	88	
Number of filicide victims	86	66	
Number of filicide cases	74	50	
Rate of filicide per 100,000	5.2 ^b^	5.9^b^	
Victims -- boys (%)	45 (52)	35 (53)	
Victims -- girls (%)	40 (47)	31 (47)	
Victims -- gender unknown (%)	1 (1)	0	
Victims -- < 24 hours, neonaticides (%)	23 (27)	5 (8)	<0.01^c^
Victim's age at time of death			
- range	0--17.8	0--17.9	
- median	2.0	3.8	
- IQR ^d^	6.7	6.1	
Number of perpetrators	74 ^e^	50	
Perpetrator -- mother (%)	57 (72)	26 (52)	<0.05^c^
Perpetrator -- father (%)	14 (18)	23 (46)	<0.001^c^
Perpetrator -- non-biological ^f ^mother (%)	1 (1)	0	
Perpetrator -- non-biological ^f ^father (%)	7 (9)	1 (2)	
Perpetrator's age at time of crime			
- range	51.9 (16--68)	33.6 (20--53)	
- mean	32.7	35.3	
- SD	10.2	8.3	

^a ^Austria's total child homicides include those that were 18 and 19 because of registering policies. In 2001 legal adulthood was lowered from 19 to 18.

^b ^Population statistics for 2001

^c ^Fisher's exact test, two-sided

^d ^Inter Quartile Range (75th percentile - 25th percentile) Victims' ages did not have normal distribution, hence median and IQR.

^e ^Two perpetrators committed two cases each, one perpetrator committed four cases, in five cases there were two perpetrators

^f ^Adoptive, foster, or stepparent

In Finland, 30% of the perpetrators were intoxicated with alcohol during their crime, in Austria 8%, the difference was significant (Fisher's exact test, p < 0.001). The perpetrator either committed or attempted suicide at the crime scene in 54% of cases in Finland, 32% in Austria (Fisher's exact test, p < 0.05). The most common methods of operation were suffocation and strangling in Austria, suffocation and shooting in Finland. Other crime scene information is presented in Table 3.

**Table 3:** Filicide in Austria and Finland 1995--2005, crime scene information

	Austria n (%)	Finland n (%)	p
Suicide - committed at crime scene	14 (18)	15 (30)	ns
Suicide - attempted at crime scene	11 (14)	12 (24)	ns
Perpetrator died at the scene	15 (20) ^a^	16 (32) ^a^	ns
Intoxicated at time of the crime	11 (14)	17 (34)	p < 0.01
Method of operation ^b^			
-- drowning	12 (17)	3 (6)	ns
-- suffocation	16 (22)	10 (20)	ns
-- shooting	6 (8)	10 (20)	ns
-- battering	4 (5)	4 (8)	ns

All tests Fisher's exact test, two sided

^a ^One perpetrator in both countries died at the scene from delivery-related causes

^b ^Percentages of methods are calculated from case number

Personality disorders were the most common (35% and 47%) psychiatric diagnostic group in both countries. Non-psychotic depression was significantly more common in Finland than in Austria (35% vs. 9%, Fisher's exact test, p < 0.05). Also substance abuse/dependency was more often diagnosed in Finland (26% vs. 2%, Fisher's exact test, p < 0.001). Other psychiatric and legal results are presented in Table 4.

**Table 4:** Filicide in Austria and Finland 1995--2005, psychiatric and legal results

	Austria **n (%)**^a^	**Finland n (%)**^a^	p
Forensic psychiatric examination	47 (83)	28 (82)	Ns
Criminally irresponsible	16 (33)	8 (25)	Ns
Psychotic disorder	13 (22)	10 (29)	Ns
- Schizophrenia/Schizoaffective disorder	5 (9)	4 (12)	Ns
- Psychotic mood disorder ^b^	6 (10)	4 (12)	Ns
Personality disorder	20 (35)	16 (47)	Ns
- Antisocial	0	1 (3)	Ns
- Borderline	2 (3)	4 (12)	Ns
Non-psychotic depressive disorder	5 (9)	12 (35)	<0.05
Substance abuse/dependency	1 (2)	9 (26)	<0.001

All tests Fisher's exact test, two sided

^a ^Percentage of living perpetrators. In Austria, 58 perpetrators survived for processes, in Finland 34

^b ^Two bipolar disorders in Austria, one in Finland, all the rest depressive disorders

There were eight non-biological parents in Austria and one in Finland (Table 2). Comparing the biological parents with the non-biological ones did not yield any significant differences. Two (22%) of the non-biological parents had committed suicide at the crime scene and one was on the run from legal officials. Six non-biological parents underwent detailed examinations, i.e. 86% of the living non-biological parents. Of these six, three had a personality disorder, two a non-psychotic mood disorder and two a substance abuse disorder. No non-biological parent suffered from a psychotic disorder.

## Discussion

To our knowledge, this was the first ever international study on filicide. It was a comprehensive and nationwide register-based study in two European countries, Austria and Finland, totalling a population of almost 14 million. There were some inherent national divergences but the similarities between the countries proved extensive enough to allow assembling data for further analyses.

In the present study, the rate of filicide was over 5 per 100,000 in both countries (Table 2), which is high in comparison to the official statistics (Table 1). This highlights the well-known problem with filicide, its hidden nature. It is a commonly acknowledged problem that the rates of child murder are underestimated due to underreporting, inaccurate coroner rulings and some bodies remaining undiscovered [29,30]. In the present study all filicide cases were studied, including the filicide-suicide cases, which increased the rate.

Globally, it is mostly men who commit homicides, but among the filicide perpetrators in the present study the gender distribution was substantially different. The proportion was almost equal in Finland, while there were more mothers than fathers in Austria, where the victims were on average the youngest. Such correspondence has also been noted earlier, i.e., that the younger the victim, the more probably is the perpetrator the mother, not the father [4,7]. Moreover, it seemed that in our data, those at highest risk of filicide were children under two years of age. Children over 10 years of age were safer (Figure 2). This concurs with previous studies [7,31].

In the present study, non-biological parents did not form a substantial or even a specific group. The number of perpatrators was, of course, too small to enable adequate statistical analyses. Yet, it may be that the definition of an actual parental relationship having had to exist, affected the results. Clearly, more study with different methodology would be necessary to describe the non-biological offenders of filicide in more detail.

The overall picture arising from our results seemed to reinforce the notion that filicide as a phenomenon is closer to suicide than it is to the average homicide. In our study, a substantial portion of the perpetrators committed suicide at the crime scene (Finland 30%, Austria 18%). When committed and attempted suicides were added up, even higher percentages were uncovered (Finland 54%, Austria 32%). This correlation between suicide and filicide has been pointed out also in previous studies [4,13].

In our study, the psychiatric diagnoses of the filicide perpetrators did not resemble those of the average homicide offender. In previous homicide studies, mood disorders have not been associated with a heightened risk of homicide [15,19]. However, depressive disorder is a clear risk factor for suicide: more than two-thirds of both suicide completers and attempters have been found to suffer major depressive episodes at the time of their suicidal act [32]. Those at risk have been reported to have more severe depressive-anxious symptomatology, as well as more impulsivity and hostility [33]. The latter two are traits of homicide offenders, too. In the present study of filicide perpetrators, the proportion of non-psychotic mood disorders was 9% and 35% and of psychotic mood disorders 10% and 12% in Austria and Finland, respectively. The higher mood disorder frequencies were found in Finland, which also ranks first between the two in suicide rates. In Europe, the 12-month prevalence of depression has been found to range from 2.6 to 9.1% [34,35]. Depressive disorders have also previously been associated with filicide and it was quite recently proposed that bipolarity should be considered when examining filicidal mothers with post-partum-onset depression, psychotic symptoms, and nonaltruistic motivation for filicide [36]. This did not, however, surface in the present study.

Alcohol abuse has long been proved to be associated with homicide [37,38]. The association is especially pronounced in Finland, where 80% of homicide offenders are intoxicated with alcohol at the time of their crime [39]. Finland is not the exception though since the association between alcohol abuse and homicide is international [19,40]. Moreover, alcohol dependence is also associated with suicide [41], while to a lesser extent, filicide perpetrators have been intoxicated during the crime [6,14,42]. Among the Finnish filicide perpetrators in the present study, 30% were intoxicated with alcohol, a similar proportion as has been found within suicide victims, 35% [43]. The intoxication percentage for Austrian filicide perpetrators registered lower. Yet, of the two, Austria ranks first in the gross consumption of alcohol per capita (Table 1). However, in Finland a mere 10% of the population drink half of the alcohol [44]. To conclude, the filicide perpetrators in the present study having not been commonly intoxicated illustrates that filicide certainly is a form of homicide separate from the rest.

In the present study, personality disorders were the most common group of diagnoses. Personality disorders, especially antisocial personality, have previously been associated with homicide [15] and also with filicide, with borderline personality prevailing [4,42,45]. In a recent comparison of filicide and other homicide perpetrators, no difference in the frequency of personality disorders in general was observed, but homicide offenders had more often antisocial personality [11].

There were some further national peculiarities in the results of the present study. Austria had most neonaticides, 27% of all cases, while Finland had 10%. Abortion has been legal in both countries for decades (Finland 1970, Austria 1975). It is performed on request in Austria but in Finland reasons need to be provided. However, socioeconomic reasons suffice. Further study is obviously needed to garner more complete knowledge on neonaticides. Furthermore, in the present study, shooting was the most common method in Finland, not in Austria. Finland ranks third in gun ownership in the world [46]. In Austria, the firearms law was amended in 1997 and the introduction of restrictive firearm legislation effectively reduced the rates of firearm suicide and homicide [47]. Indeed, an association between household firearm ownership and rates of suicide and homicide has been established [48,49]. The question arises, whether or not the shooting filicides in Finland could have been prevented with stricter legislation. Gun control legislation and the percentage of households with guns have been discussed previously in reference with the prevention of filicide [31,50]. To summarise, differences between the two countries emerged which suggested a need to find other grouping factors besides nationality. Yet, there are sufficient similarities between the countries to enable the merging of the national data for further studies, which are clearly needed.

## Strengths and limitations

Both countries maintain highly reliable registers and an appreciable portion of homicide offenders go through a forensic psychiatric examination as part of the trial procedure. These examinations are lengthy and thorough. Because of the nature of the crime, an even larger proportion of filicide perpetrators are examined than average homicide perpetrators. In Austria and Finland, quite equal proportions of filicide perpetrators, representing a reasonably high percentage of all living perpetrators (82 and 83%), underwent a forensic psychiatric examination. Thus, we received quite comprehensive data on the psychiatric illnesses of living filicide perpetrators.

It seems that the two European countries are similar enough for joint inspection of the phenomenon of filicide. Child murder, especially in the first year of life, has been revealed as deeply embedded in the societies in which they occur [51]. The societal status of women can be judged as similar enough for comparison in both countries. The mean age of women at first live birth in 2005 was 27 in Austria and 28 in Finland [52,53]. Yet, cultural differences might have had some effect on methodology.

The fact that this was a comprehensive nationwide and international study in two European countries is a definite strength. No such study has been published before. We achieved a collection of more widespread data than with national data only, which enhances generalisability of results as well as the possibility of further analyses. However, retrospective register based studies have their obvious limitations. Furthermore, even with such comprehensive data some cases, mainly neonaticide cases, might have been overlooked. Moreover, since the results arise from European countries, their generalization to, e.g., the US is problematic. However, one of the invaluable additions of the present study is the link of filicide-suicide, which has been commonly excluded in filicide studies.

## Conclusion

Filicide is a distinct type of homicide that demands special consideration. Suicidality is undoubtedly associated with filicide. The present study demonstrated that the data of the two countries can and should indeed be joined more fully for further common analyses. Perhaps there exist several types of offences and perpetrators with a specific constellation of current and background variables of which we are presently unaware. With this international study we will be able to perform further analyses with the aim of achieving a detailed description of putative new subgroups, and, most importantly, recommendations for preventive actions.

## Competing interests

Dr. Eronen has received speakers honoraria from Bristol-Myers Squibb, Astra Zeneca, Orion, and Novartis. Dr. Klier has received speakers honoraria from Wyeth Lederle Pharma, Lundbeck, Eli Lilly and Janssen-Cilag Pharma. These are single occasions with minor economic significance. All other authors report no competing interests.

## Authors' contributions

HP contributed to original idea, conception, design, and acquisition of data, analyzed and interpreted data, and served as first author. SA contributed to conception, design, and acquisition of data, analyzed and interpreted data, and served as second author. MPA contributed to original idea, conception, design, and participated in the writing process JYC contributed to conception, design, and participated in the writing process ME contributed to conception, design, and participated in the writing process. CK contributed to conception, design, and participated in the writing process. EK contributed to conception, design, and participated in the writing process. GW-H contributed to conception, design, and acquisition of data, analyzed and interpreted data, and participated in the writing process. All authors read and approved the final manuscript.

## Pre-publication history

The pre-publication history for this paper can be accessed here:

http://www.biomedcentral.com/1471-244X/9/74/prepub
